# Phytochemical Studies on Two Unexplored Endemic Medicinal Plants of India, *Barleria terminalis* and *Calacanthus grandiflorus*

**DOI:** 10.3389/fphar.2021.817885

**Published:** 2022-01-17

**Authors:** Shreedhar S. Otari, Suraj B. Patel, Manoj M. Lekhak, Savaliram G. Ghane

**Affiliations:** ^1^ Plant Physiology Laboratory, Department of Botany, Shivaji University, Kolhapur, India; ^2^ Angiosperm Taxonomy Laboratory, Department of Botany, Shivaji University, Kolhapur, India

**Keywords:** biological activities, endemic, HPLC, LC-MS, multivariate analysis, phytochemicals

## Abstract

*Barleria terminalis* Nees and *Calacanthus grandiflorus* (Dalzell) Radlk. are endemic medicinal plants of the Western Ghats of India. The aim of the present research work was to investigate phytochemical profile, potent bioactives using RP-HPLC, LC-MS and GC-MS and to evaluate their bioactivities. Acetone was found to be the best extraction medium for separating phytochemicals. Similarly, acetone and methanol extracts exhibited potential antioxidant properties. Ethanol extract of *B. terminalis* stem showed potent acetylcholinesterase (AChE) (89.10 ± 0.26%) inhibitory activity. Inhibition of α-amylase (36.96 ± 2.96%) activity was observed the best in ethanol extract of *B. terminalis* leaves and α-glucosidase inhibitory activity (94.33 ± 0.73%) in ethanol extract of *C. grandiflorus* stem. RP-HPLC analysis confirmed the presence of several phenolic compounds (gallic acid, hydroxybenzoic acid, vanillic acid, chlorogenic acid and coumaric acid) and phenylethanoid glycoside (verbascoside). The highest phenolics content were observed in *B. terminalis* (GA (4.17 ± 0.002), HBA (3.88 ± 0.001), VA (4.54 ± 0.001), CHLA (0.55 ± 0.004) mg/g DW, respectively). Similarly, LC-MS and GC-MS revealed the presence of phenolics, glycosides, terpenes, steroids, fatty acids, etc. Moreover, positive correlation between studied phytochemicals and antioxidants was observed in principal component analysis. Based on the present investigation, we conclude that *B. terminalis* and *C. grandiflorus* can be further explored for their active principles particularly, phenylethanoid glycosides and iridoids and their use in drug industry for pharmaceutical purposes.

## 1 Introduction

Acanthaceae Juss. is a large family comprising about 4,000 species and 220 genera over the globe. It comprises annual, perennial, herbs, shrubs or trees found in tropical, subtropical and a few in temperate regions (Kavitha et al., 2016). *Barleria terminalis* Nees and *Calacanthus grandiflorus* (Dalzell) Radlk. are the endemic plants of the Western Ghats of India (Tripp et al., 2013; Patil et al., 2019). The genus *Barleria* L. contains 285 species found in Asia and Africa, with the majority of its diversity found in tropical eastern and southern Africa (Darbyshire et al., 2019). In India, the genus is represented by 30 species (Patil et al., 2020). Extensive literature survey showed that *Barleria* is medicinally important genus and locally called as Vajradanti (Banerjee et al., 2012). The plant is widely used in the treatment of human diseases like anemia, toothache, cough, fever, asthma, bronchitis, diabetes, insect bites and inflammations (Banerjee et al., 2012; Sudheer and Praveen, 2021). Aerial parts and leaves of *Barleria* are known to be a vital source of iridoids (Amoo et al., 2011; Sudheer and Praveen, 2021). The leaves of the plant are known for flavonoids, saponins, sterols, tannins, and terpenoids. Similarly, flowers contain flavonoids and neohesperidoside. In the same way, aerial part contains balarenone, terpenoid, barlerinoside, saponins, flavonoids, phenolics, tannins, steroids, carbohydrates, phytosterols, acetyl-barlerin, β-sitosterol, iridoids and lupulinoside (Ata et al., 2009). This plant contains numerous medicinal properties (Sudheer and Praveen, 2021). On the other hand, there are no reports on the phytochemical or medicinal potential of *C. grandiflorus*.

So keeping in view the therapeutic potential of *Barleria* and *Calacanthus*, leaves and stem extracts of *B. terminalis* and *C. grandiflorus* were prepared for the first time utilizing a variety of solvents. The present study was designed to explore the phytochemicals and biological potential of both the species (antioxidant, anti-acetylcholine esterase and antidiabetic**)** as well as to identify and quantify phenolics and phenylethanoid glycoside (verbascoside) using RP-HPLC. Furthermore, LC-MS and GC-MS were used to screen potent secondary metabolites present in the plants. To the best of our knowledge, this is the first study to look into the potential benefits of the medicinal plants, *B. terminalis* and *C. grandiflorus*.

## 2 Materials and Methods

### 2.1 Collection of Plant Materials and Extract Preparation

*B. terminalis* was collected from Lead Botanical Garden, Shivaji University, Kolhapur, Maharashtra (N 16°40.546’, E 074°15.337’). *C. grandiflorus* were collected from Kumbhavade ghat, Sindhudurg district, Maharashtra (N 16°31.51.9′, E 73°50′01.4′). Both the specimens were deposited in SUK herbarium (Voucher No. SSO 001, SSO 030). Plant material was oven dried for 72 h at 60°C, ground into fine powder, and extraction was done by continuous shaking of finely ground powder (5 g) in respective solvents (30 ml each). Extracts were then centrifuged (5,000 rpm), concentrated and dissolved in 5 ml respective solvent. Prior to analysis, extracts were filtered through 0.2 µm nylon filter (HiMedia, India), used in experiment and stored at 4°C.

### 2.2 Phytochemical Analysis

#### 2.2.1 Estimation of Total Phenolic Content, Total Flavonoid Content, Total Tannin Content

TPC was determined as per the method adopted by Patel and Ghane (2021) with some minor modifications. Results were expressed as mg tannic acid equivalent (TAE)/g extract. TFC was quantified according to method described by Sakanaka et al. (2005) and TTC was estimated as per Patel and Ghane (2021) with minor modifications. Catechin was used as standard (mg/ml) and results were expressed in mg catechin equivalent (CE)/g extract.

#### 2.2.2 Estimation of Proanthocyanidins and Total Iridoid Content

Total proanthocyanidins were calculated according to the method described by Fawole et al. (2009) with minor modifications. Proanthocyanidins content was expressed as % per g DW and calculated by the following formula:

$$Proanthocyanidins\;\left(\%\;dry\;matter\right)=\frac{A550nm\;\times \;78.26\;\times \;Dilution\;factor\;\;}{\%\;Dry\;matter}\times \;100$$

TIC was estimated according to method described by Levieille and Wilson (2002) with minor modifications. Results were expressed as mg harpagoside equivalent (HE)/g extract.

### 2.3 Determination of Antioxidant Potential

#### 2.3.1 DPPH Scavenging Assay, FRAP Assay, ABTS Assay, Metal Chelating Assay, Phosphomolybdenum Assay

DPPH radical scavenging activity was determined as per earlier method (Attar and Ghane, 2021). The activity was expressed as mg ascorbic acid equivalent (AAE)/g extract. The FRAP method proposed by Benzie and Strain (1996) was adopted. FRAP values were indicated as mg Fe (II) equivalent/g extract. ABTS radical activity was evaluated according to Patel et al. (2018) with some minor modifications. Activity was expressed as mg trolox equivalent (TE)/g extract. Metal chelating activity was calculated as per Patel and Ghane (2021) and the activity expressed as mg EDTA equivalent (EE)/g extract. Phosphomolybdenum assay was performed according to method described by Prieto et al. (1999). Results were depicted as mg of ascorbic acid equivalent (AAE)/g extract.

#### 2.3.2 Acetylcholinesterase Inhibitory and Anti–diabetic Activities

AChE inhibitory activity was examined according the method of Ghane et al. (2018) with some minor modifications. Galanthamine hydrobromide was used as standard, and results were expressed in percentage. Inhibition of *α*-amylase and *α*-glucosidase was evaluated as per our earlier protocol (Ghane et al., 2018). Acarbose was used as a positive control and activity was evaluated on a percent basis.

### 2.4 Analysis of Phenolics, Verbascoside and Other Metabolites by RP-HPLC, HR-LC-MS and GC-MS

#### 2.4.1 Preparation of Samples and Standard Solutions

Finely ground powder (500 mg) of *B. terminalis* and *C. grandiflorus* (leaves and stem) was extracted by using 10 ml methanol in an ultrasound bath. Homogenate was centrifuged, and supernatant collected, condensed and volume adjusted to 1 ml. Prior to analysis, the extract was filtered using 0.2 µm nylon filter (HiMedia, India). Different concentrations of standard solutions (20–100 μg/ml) were prepared and used to plot calibration curve using RP-HPLC.

#### 2.4.2 RP-HPLC Analysis of Phenolics

HPLC apparatus consisted of quaternary pump, autosampler and UV detector (UV 2070) (Jasco, Japan, Model no. LC-2000 plus). Separation was performed using Hiber C18 column (5 μm, 250–4.6 mm). Mobile phase consisted of water: acetonitrile: glacial acetic acid (90:5:5), flow rate was 0.9 ml/min and 20 μl injection volume (Patel and Ghane, 2021). Sample peaks were monitored at 280 nm with 60 min as run time. Phenolic content was determined by comparing with standards and expressed as milligram per gram of dry weight (mg/g DW).

#### 2.4.3 RP-HPLC Analysis of Verbascoside

RP-HPLC analysis of verbascoside was carried out using the same instrumentation as specified above. Separation of compound was achieved using methanol and water (90:10) as mobile phase with 1 ml/min flow rate and 20 μl injection volume (Dhakulkar et al., 2005). The peak was monitored at 320 nm with 30 min as run time. Experiments were performed in triplicates for assessing suitability of system and amount of verbascoside expressed as µg/g DW.

#### 2.4.4 Identifications of Major Metabolites by LC-MS

LC-MS analysis was done by using HPLC-ESI-MS-NEG-PHENOMENEX in negative ionization mode. System was equipped with binary pump, auto sampler, thermostated column compartment and iFunnel quadrapole time-of-flight spectrometer (Q-TOF). Zorbax eclipse C18 column (4.6 × 250 mm, 5 μm particle size) was used for compounds separation at 25°C temperature. In the present study, 0.1% (v/v) formic acid (A) and acetonitrile (B) was used in gradient elution. Gradient was initiated at 80% A and 20% B to 30% B (after 10 min), followed by 40% B (40 min), 60% B (60 min) and 90% B (80 min) and finally returned to the initial conditions. Solvent system B was injected with flow rate of 0.8 ml/min. Mass spectrometer was operated in range of 100–1,000 m/z. N_2_ gas was used as a nebulizer. Drying gas flow rate was 8 L/min at 325°C and nebulizer gas at 25 psi with fragmentor voltage 150 V (Patel and Ghane, 2021). For data analysis, mass hunter qualitative analysis software package (Agilent Technologies) was used. Detected compounds were validated on the basis of molecular formula, molecular mass, retention time and m/z ratio. For the authentication of compounds, details were compared with available literature and Metline personal metabolites database.

#### 2.4.5 Identifications of Major Metabolites by GC-MS

GC-MS analysis was performed on Model QP 2010 series, Shimadzu, Tokyo, Japan, equipped with AOC-20i auto sampler and RTX-1 fused silica capillary column (30 m length, 0.25 mm id, and 0.25 µm thickness). Helium gas (purity 99.99%) was used as a carrier gas at a flow rate of 1.5 ml/min. The injector temperature was fixed at 280°C and samples were injected through split injection mode. The column oven program was set at 50°C for 2 min, then increased to 28°C with the rate of 10°C/min. Ion source temperature was applied to 230°C and interface temperature was set to 250°C. The mass range from 36 to 800 m/z was scanned at a rate of 3.0 scans/s. The total run time of GC-MS system was 36 min (Patel and Ghane, 2021). Compounds were identified by comparison with authentic spectra obtained from GC-MS library (NIST 11).

### 2.5 Statistical Analysis

All analyses were performed in triplicates and values are represented as average, with standard error. Data obtained from the experiments were subjected to one-way analysis of variance and significant differences between mean values were determined by Duncan’s multiple range test (*p* ≤ 0.05) using SPSS software ver. 16. Data derived from the studied phytochemicals and antioxidant activities from different extracts were subjected to Principal Component Analysis (PCA) (Minitab software ver. 19).

## 3 Results

### 3.1 Phytochemical Analysis

In phytochemicals such as TPC, TFC, TTC, proanthocyanidins and TIC contents were determined and results are represented in Table 1. Among all the samples, *Calacanthus grandiflorus* leaves extract showed comparatively higher yield as compared to the stem extracts that ranged from 2.36 to 5.69%.

**TABLE 1:** Extract yield, total phenolics content (TPC), total flavonoids content (TFC), total tannins content (TTC), proanthocyanidins and total iridoids content (TIC) of different solvent extracts of *B. terminalis* and *C. grandiflorus*.

Species	Plant part	Solvents	Yield (%)	TPC^α^	TFC^β^	TTC^β^	Proanthocyanidins^λ^	TIC^ƴ^
*Barleria terminalis*	Leaves	Acetone	2.62	158.7 ± 4.07^a^	144.10 ± 3.42^a^	200.60 ± 11.32^c^	4.89 ± 0.61^c^	3.28 ± 0.4^b^
		Ethanol	4.14	94.36 ± 0.60^c^	98.24 ± 1.85^b^	102.42 ± 9.46^e^	0.92 ± 0.16^defg^	0.15 ± 0.0^g^
		Methanol	2.84	105.63 ± 0.22^b^	103.23 ± 6.94^b^	32.72 ± 1.04^h^	1.09 ± 0.23^def^	5.77 ± 0.4^a^
		water	5.48	40.07 ± 1.78^f^	25.4 ± 0.06^efg^	81.81 ± 4.81^fg^	0.074 ± 0.013^g^	2.68 ± 0.1^bc^
	Stem	Acetone	0.70	60.60 ± 2.09^e^	35.14 ± 3.60^de^	74.54 ± 11.32^g^	5.66 ± 0.26^bc^	0.53 ± 0.0^g^
		Ethanol	2.21	18.01 ± 0.28^gh^	27.22 ± 1.44^ef^	17.57 ± 9.46^ij^	1.24 ± 0.30^de^	0.18 ± 0.0^g^
		Methanol	1.98	15.93 ± 0.61^ghi^	20.76 ± 1.90^fg^	6.66 ± 1.04^j^	0.96 ± 0.23^defg^	2.68 ± 0.2^bc^
		Water	3.42	13.78 ± 0.24^ih^	20.76 ± 3.66^efg^	4.24 ± 4.81^j^	0.16 ± 0.02^fg^	3.34 ± 0.1^b^
*Calacanthus grandiflorus*	Leaves	Acetone	2.36	81.74 ± 7.09^d^	81.93 ± 1.38^c^	290.30 ± 1.04^a^	6.51 ± 0.08^b^	0.42 ± 0.1^g^
		Ethanol	2.74	24.7 ± 6.69^g^	29.88 ± 1.79^e^	124.24 ± 2.64^dg^	1.78 ± 0.45^d^	2.01 ± 0.1^de^
		Methanol	2.46	16.11 ± 1.26^ghi^	24.56 ± 0.52^fg^	70.90 ± 1.21^g^	1.10 ± 0.26d^ef^	1.40 ± 0.0^f^
		water	5.69	33.88 ± 0.38^f^	14.77 ± 1.85^efg^	90.30 ± 1.21^ef^	0.07 ± 0.0019^g^	2.26 ± 0.1^cde^
	Stem	Acetone	1.02	105.33 ± 2.60^b^	40.53 ± 6.12^d^	248.48 ± 4.24^b^	8.74 ± 0.67^a^	2.92 ± 0.0^bc^
		Ethanol	3.22	18.38 ± 0.28^g^	22.43 ± 3.83^fg^	52.12 ± 0.60^h^	0.80 ± 0.18^efg^	0.25 ± 0.00^g^
		Methanol	3.54	9.98 ± 5.27^hi^	19.70 ± 1.13^fg^	35.75 ± 1.60^h^	0.27 ± 0.05^efg^	2.63 ± 0.1^bcd^
		Water	3.89	7.10 ± 0.38^i^	20.50 ± 3.52^efg^	21.81 ± 4.19^efhi^	0.063 ± 0.006^g^	1.69 ± 0.6^ef^

Values are means of three replicate determinations ±standard error. Mean values in the same column with different alphabets showed statistically significant differences (*p* ≤ 0.05) according to Duncan’s multiple range test. ^α^(mg TAE/g extract), ^β^(mg CE/g extract), ^λ^(%/g DW), ^ƴ^(mg HE/g extract).

In the case of *B. terminalis,* the highest TPC and TFC content were found in acetone leaves extract (158.76 ± 4.07 mg TAE/g extract and 144.10 ± 3.42 mg CE/g extract, respectively). TTC ranged from 4 to 290 mg CE/g extract where aqueous stem extract possessed the lowest tannin content (4.24 ± 1.21 mg CE/g extract). Similarly, total iridoid content was recorded the highest in methanol extract of leaves (5.77 ± 0.4 mg HE/g extract) and the lowest was found in ethanol extract of stem (0.18 ± 0.0 mg HE/g extract) (Table 1).

In *C. grandiflorus*, aqueous extract of stem had the least content (7.10 ± 0.38 mg TAE/g extract) of TPC. In the same way, the lowest TFC was exhibited in aqueous leaves extract (14.77 ± 1.85 mg CE/g extract). Acetone extract of leaves revealed promising tannin content (290.30 ± 1.04 mg CE/g extract). Moreover, proanthocyanidins content ranged from 0.06 to 9%/g DW and acetone extract of stem had the highest proanthocyanidins (8.74 ± 0.67%/g DW) whereas water extracts exhibited the least (0.063 ± 0.006%/g DW) content (Table 1).

### 3.2 Antioxidant Analysis

**FIGURE 1:**
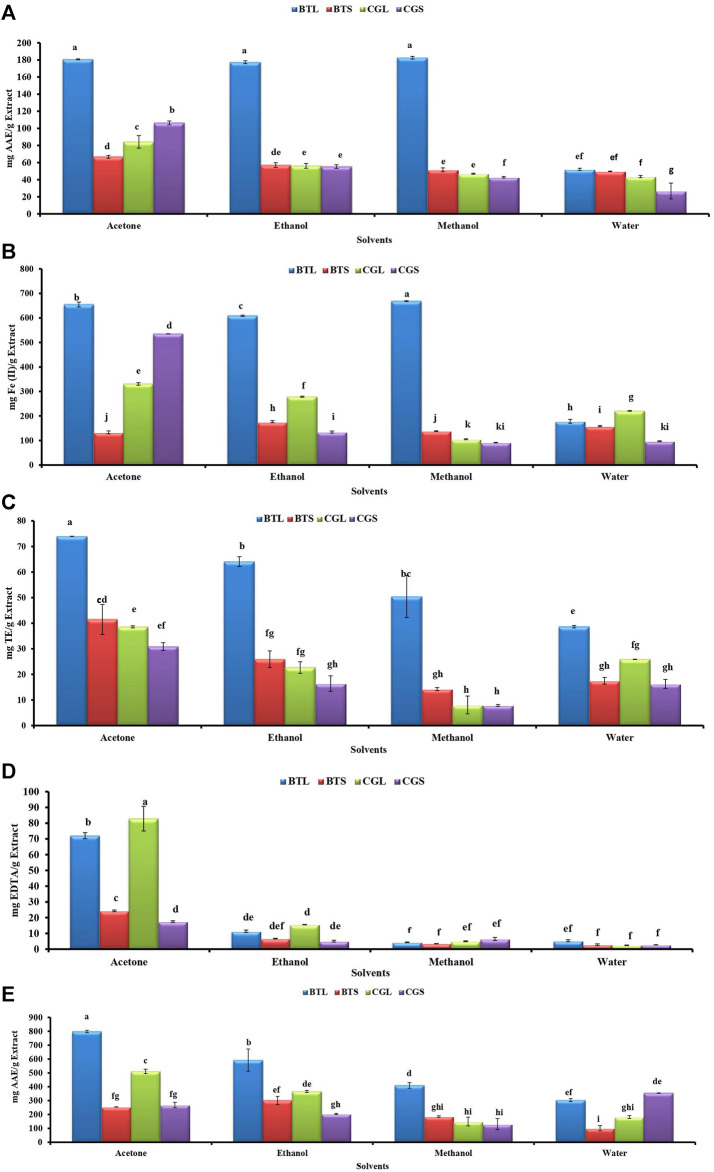
Antioxidant activities from the different extracts of *B. terminalis* and *C. grandiflorus* **(A)** DPPH radical scavenging activity, **(B)** FRAP activity, **(C)** ABTS + radical scavenging activity, **(D)** Metal chelating activity, **(E)** Phosphopmolybdenum reduction activity. Note—All figures indicate different extracts from leaves and stems of BTL—*B. terminalis* leaves, BTS—*B. terminalis* stem and CGL—*C. grandiflorus* leaves and CGS—*C. grandiflorus* stem. According to DMRT, Bars having different alphabets showed statistically significant differences (*p* ≤ 0.05).

Antioxidant activities like DPPH, FRAP, ABTS, MCA and PMA from different leaves and stem extracts of both the species are depicted in Figure 1. In *B. terminalis*, methanol extract of leaves showed promising DPPH scavenging and FRAP activity (182.59 ± 1.57 mg AAE/g extract and 668.24 ± 2.04 mg Fe(II)/g extract, respectively) (Figures 1A,B). Similarly, ABTS activity was noted the highest in the acetone extract of leaves (73.97 ± 0.04 mg TE/g extract) (Figure 1C). Acetone extract of leaves (797.61 ± 8.71 mg AAE/g extract) showed maximum PMA whereas the aqueous extract of stem exhibited the lowest activity (101.5 ± 18.95 mg AAE/g extract) (Figure 1E).

In *C. grandiflorus*, DPPH activity was observed the least in the water extract of stem (26.79 ± 9.30 AAE/g extract) (Figure 1A). Methanol extract of stem exhibited the lowest FRAP activity (91.33 ± 0.94 mg Fe (II)/g extract) (Figure 1B). The lowest ABTS activity (7.85 ± 0.37 mg TE/g extract) was registered with methanol extract of stem (Figure 1C). Antioxidant activity in terms of MC was found superior in *C. grandiflorus.* The acetone extract of leaves exhibited the highest MC activity (82.83 ± 7.79 mg EDTA/g extract) whereas the least activity was observed in the water extracts (2.42 ± 0.14 mg EDTA/g extract) (Figure 1D).

### 3.3 Chemometric Analysis

**FIGURE 2:**
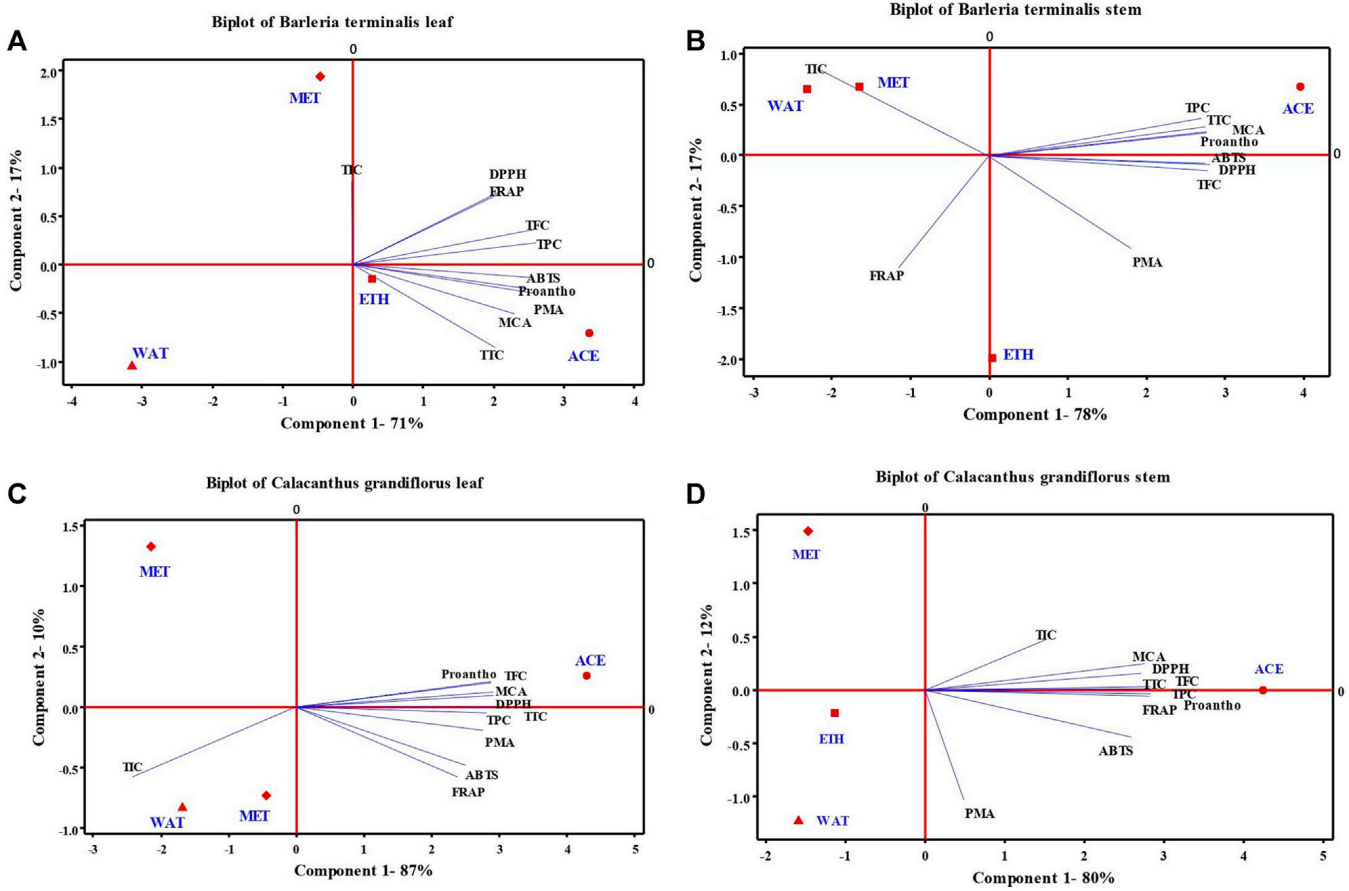
Principal component analysis (scores and loading plots, biplot) depends on several phytochemical compounds analyzed in four different leaves and stem extract of *B. terminalis* **(A,B)** and C. *grandiflorus* **(C,D)** with their antioxidant activities (DPPH, ABTS, FRAP, PMA, and MCA). TPC, total phenolic content; TFC, total flavonoid content; TTC, total tannin content; Proantho, Proanthocyanidins content; TIC, total iridoid content.

The PCA was performed to understand the relationship between the phytochemicals (TPC, TFC, TTC, proanthocyanidins and TIC) and antioxidant activities (DPPH, FRAP, ABTS, PMA, and MCA) studied from acetone, ethanol, methanol and water extracts of leaves and stem of *B. terminalis* and *C. grandiflorus* (Figure 2). From Figures 2A,B, it was confirmed that only acetone along with ABTS, proanthocyanidins, PMA, MCA and TTC occupied positive plane of component 1 in leaves; while in stem with TPC and TTC occupied positive plane of both components. PCA analysis of *C. grandiflorus* leaves and stem denoted 97 and 93% total variability respectively, out of which component 1 contributed for 87 and 80% variability. Similar to *B. terminalis* leaves, TPC, TFC, TTC, DPPH, ABTS, FRAP, PMA, MCA and proanthocyanidins enjoyed the positive plane of component 1 and exhibited largest distribution with the coefficients 0.323, 0.331, 0.339, 0.335, 0.288, 0.275, 0.317, 0.336, and 0.331, respectively (Figure 2C). PCA of stem extract of *C. grandiflorus* showed that all tested parameters enjoyed positive plane of component 1. Among the solvents studied, only acetone and methanol represented positive planes on component 1 and 2, respectively. Variables exhibited the largest distribution with the coefficients 0.353, 0.352, 0.353, 0.339, 0.323, 0.353, 0.061, 0.345, 0.353, and 0.189, respectively (Figure 2D).

### 3.4 Anti-Acetylcholinesterase and Anti-Diabetic Activities

Anti-acetylcholinesterase (AChE) and anti-diabetic activities of different solvent extracts of leaves and stem of *B. terminalis* and *C. grandiflorus* were performed and the results are depicted in Table 2. Anti-diabetic activity was analyzed by using *α*-amylase and *α*-glucosidase inhibition. In *B. terminalis*, maximum *α*-amylase inhibition was found in the ethanol extract of leaves (36.96 ± 2.96%) and the least *α*-glucosidase inhibitory (2.38 ± 2.08%) activity in the aqueous extract of leaves. The ethanol extract of stem showed the highest AChE inhibition (89.10 ± 0.26%) while the aqueous extract the least (9.12 ± 0.26%) (Table 2). Similarly, methanolic extract of leaves of *C. grandiflorus* exhibited the lowest α-amylase (14.8 ± 0.37%) inhibitory activity whereas the ethanol extract of the stem exhibited the highest *α*-glucosidase inhibitory (94.33 ± 0.73%) activity (Table 2).

**TABLE 2:** *α*-Amylase, *α*-glucosidase, and acetyl cholinesterase inhibitory activities of leaves and stem extracts of *B. terminalis* and *C. grandiflorus*.

Species	Plant part	Solvent	α-Amylase^α^ inhibitory activity (%)	α-Glucosidase^α^ inhibitory activity (%)	Acetyl cholinesterase^β^ inhibitory activity (%)
*Barleria terminalis*	Leaves	Acetone	20.02 ± 1.83^cd^	3.94 ± 1.23^ef^	12.34 ± 6.17^gh^
		Ethanol	36.96 ± 2.96^a^	26.82 ± 1.58^d^	44.01 ± 10.46^d^
		Methanol	31.27 ± 1.31^ab^	28.44 ± 2.32^d^	22.00 ± 7.78^fg^
		Water	18.28 ± 3.38^cd^	2.38 ± 2.08^f^	ND
	Stem	Acetone	17.05 ± 3.67^d^	50.91 ± 0.14^c^	61.72 ± 0.80^bc^
		Ethanol	34.37 ± 0.0^a^	62.55 ± 0.85^b^	89.10 ± 0.26^a^
		Methanol	20.16 ± 2.54^cd^	87.11 ± 0.47^a^	26.03 ± 2.68^efg^
		Water	18.56 ± 2.070^cd^	4.94 ± 0.35^e^	9.12 ± 0.26^gh^
*Calacanthus grandiflorus*	Leaves	Acetone	26.14 ± 0.047^bc^	4.05 ± 1.64^ef^	19.05 ± 2.14^fg^
		Ethanol	32.1 ± 1.27^ab^	31.67 ± 2.85^d^	77.83 ± 4.56^ab^
		Methanol	14.8 ± 0.37^d^	12.41 ± 3.35^e^	32.20 ± 7.24^ef^
		Water	23.83 ± 3.67^c^	4.58 ± 0.94^ef^	ND
	Stem	Acetone	17.43 ± 2.35^d^	52.79 ± 3.02^c^	87.76 ± 9.12^a^
		Ethanol	25.85 ± 2.58^bc^	94.33 ± 0.73^a^	77.83 ± 2.95^b^
		Methanol	19.08 ± 2.58^cd^	64.11 ± 9.11^b^	34.88 ± 10.46^ef^
		Water	18.98 ± 3.62^cd^	3.47 ± 1.64^ef^	ND

^α^ % inhibition at standard acarbose at 100 μg – 36.84%, ^β^acetylcholine esterase inhibition at standard galanthamine (3 μg)—32.41%, ND- Not Detected. Values are means of three replicate determinations ±standard error. Mean values in the same column with different alphabets showed statistically significant differences (*p* ≤ 0.05) according to Duncan’s multiple range test (DMRT).

### 3.5 Detection of Phenolics, Verbascoside and Other Bioactives

**FIGURE 3:**
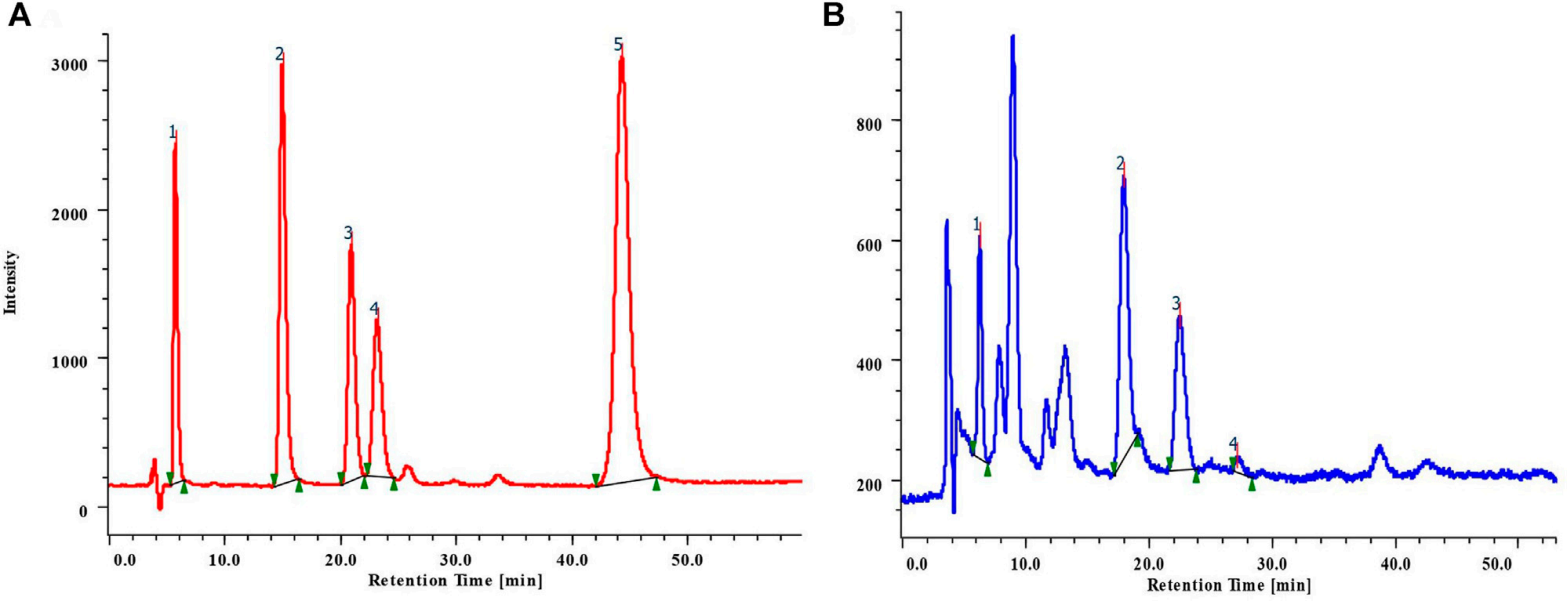
**(A)** RP-HPLC chromatogram of mixture of standard phenolic compounds 1) GA; 2) HBA; 3) VA; 4) CHLA; 5) COA; **(B)** Phenolics detected from the leaves extract of *B. terminalis*.

Results suggested that phenolics were present in the methanolic leaves and stem extracts of *B. terminalis* and *C. grandiflorus*. Five phenolic compounds, *viz*. gallic acid (GA) (tR 6.0), hydroxybenzoic acid (HBA) (tR 15.71), vanillic acid (VA) (tR 21.62), chlorogenic acid (CHLA) (tR 23.82), and coumaric acid (COA) (tR 44.87) were identified and quantified (Figure 3A; Table 3). The methanolic extract of stem of *B. terminalis* showed the highest GA content (4.17 ± 0.002 mg/g DW) whereas the lowest was observed in leaves (1.61 ± 0.003 mg/g DW). HBA content (3.88 ± 0.001 mg/g DW) was recorded maximum in leaves (Figure 3B). VA was found maximum in the leaves (4.54 ± 0.001 mg/g DW) and the minimum in the stem (0.45 ± 0.003 mg/g DW). CHLA was detected in very low quantity as compared to other phenolics. In all tested samples, the highest CHLA was recorded in the stem extract (0.55 ± 0.004 mg/g DW) (Table 3).

**TABLE 3:** HPLC analysis of phenolics from the leaves and stem extracts of *B. terminalis* and *C. grandiflorus*.

Plant samples	Phenolic compounds				
	GA^α^	HBA^α^	VA^α^	CHLA^α^	COA^α^
	(tR 6.0)	(tR 15.71)	(tR 21.62)	(tR 23.82)	(tR 44.87)
BTL	1.61 ± 0.003^d^	3.88 ± 0.001^a^	4.54 ± 0.001^a^	0.39 ± 0.003^b^	ND
BTS	4.17 ± 0.002^a^	ND	0.45 ± 0.003^b^	0.55 ± 0.004^a^	1.73 ± 0.002^b^
CGL	2.61 ± 0.002^c^	0.003 ± 0.036^b^	0.41 ± 0.001^c^	ND	0.43 ± 0.002^c^
CGS	3.26 ± 0.001^b^	ND	0.52 ± 0.004^d^	0.22 ± 0.002^c^	52.82 ± 0.002^a^

ND – Not detected, BTL – B. terminalis leaves, BTS – B. terminalis stem, CGL – C. grandiflorus leaves, CGS – C. grandiflorus stem. Values are means of three replicate determinations ±standard error. Mean values in the same column with different alphabets showed statistically significant differences (*p* < 0.05) according to DMRT. ^α^ (mg/g DW).

In the case of *C. grandiflorus*, significant amount of GA content was found in the stem (3.26 ± 0.001 mg/g DW) followed by the leaves (2.61 ± 0.002 mg/g DW) extract. VA was found maximum in the stem (0.52 ± 0.004 mg/g DW) and minimum in the leaves (0.41 ± 0.001 mg/g DW) extract. CHLA was observed to be the lowest in the stem (0.22 ± 0.002 mg/g DW) extract. The highest content of COA was reported in the stem (52.82 ± 0.002 mg/g DW) extract (Table 3).

**FIGURE 4:**
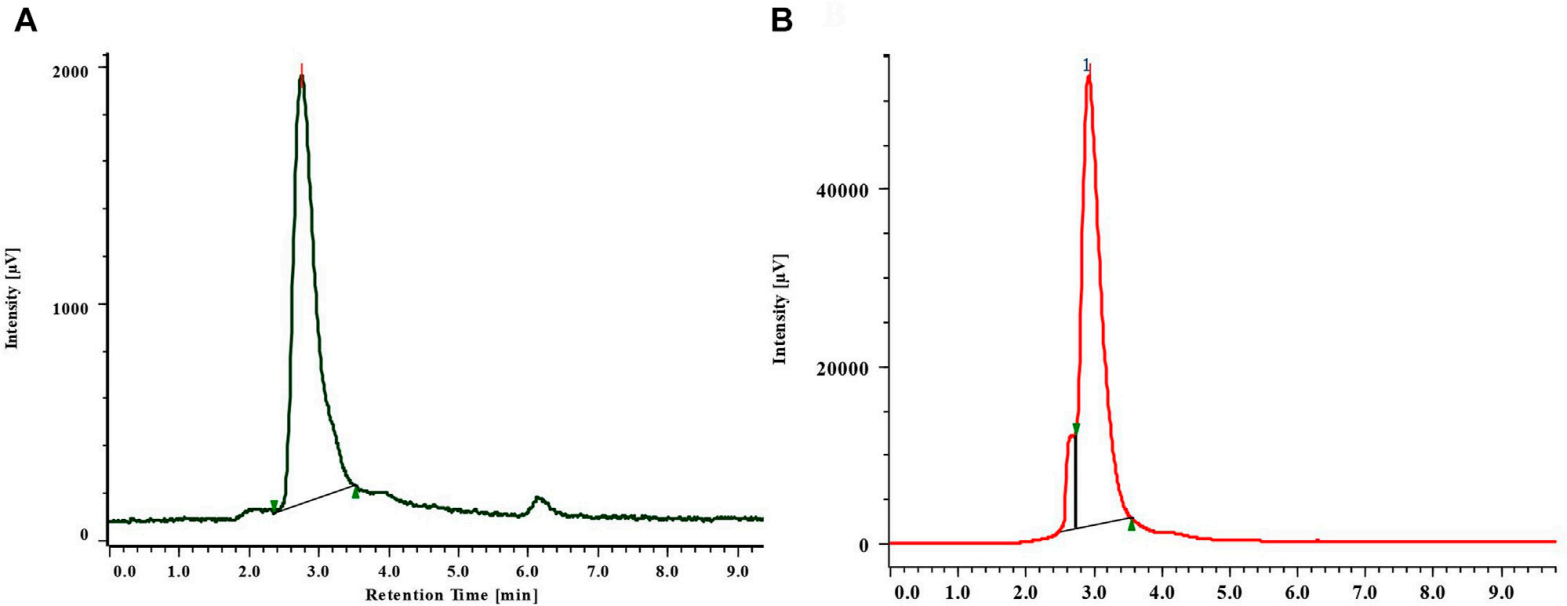
RP-HPLC chromatogram of standard **(A)** and verbascoside from the leaves of *B. terminalis* **(B).**

**FIGURE 5:**
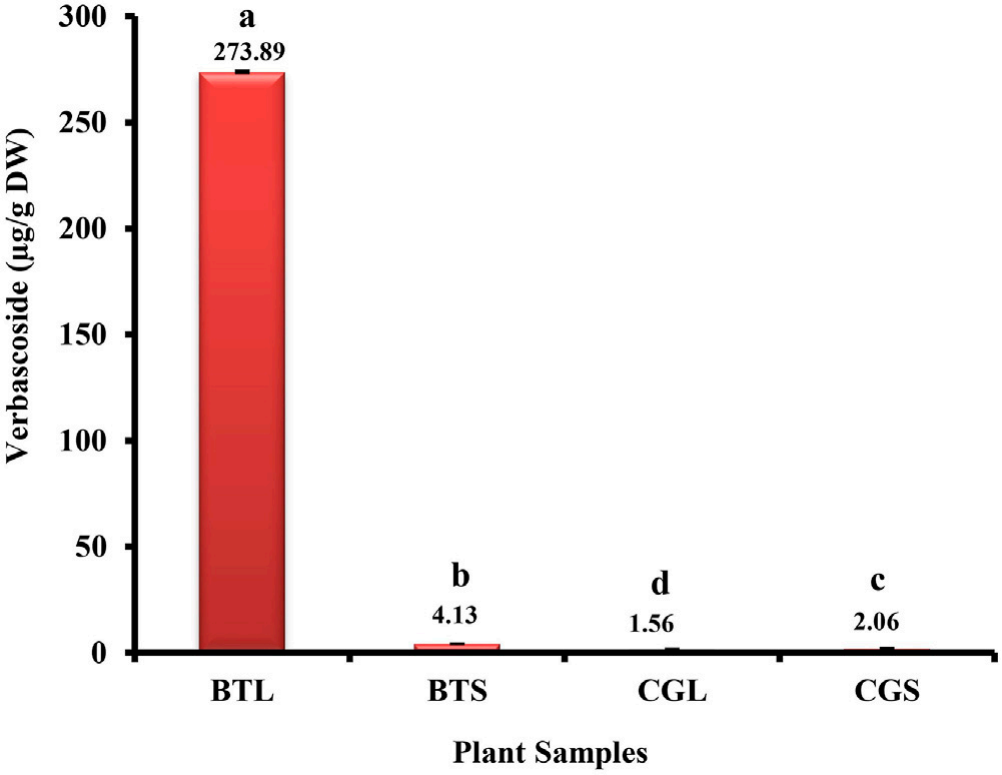
HPLC analysis of verbascoside from *B. terminalis* and *C. grandiflorus*. BTL—*B. terminalis* leaves, BTS—*B. terminalis* stem, CGL—*C. grandiflorus* leaves, CGS—*C. grandiflorus* stem.

**FIGURE 6:**
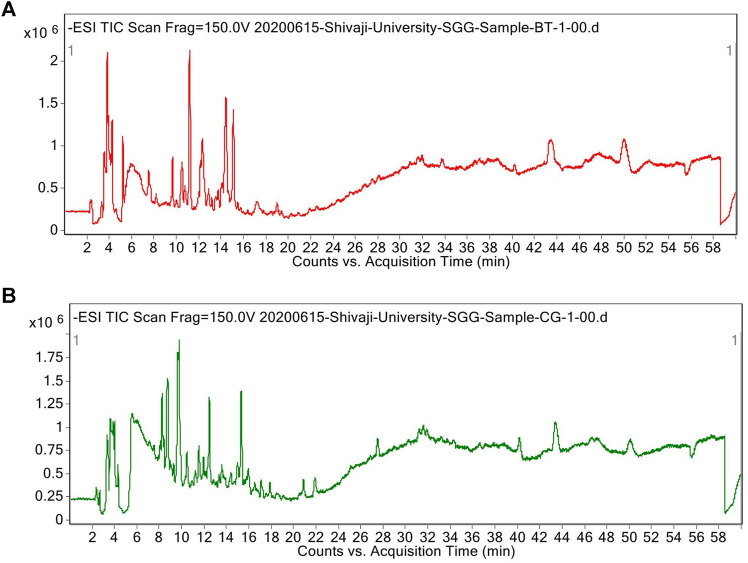
Total ion chromatograms (TICs) of methanol extract of *B. terminalis* leaves **(A)** and *C. grandiflorus* leaves **(B)**.

Verbascoside was detected and quantified from the methanolic extracts of both the species (Figure 4 and Figure 5). All the plant parts showed remarkable quantity of verbascoside, in which the highest content was found in *B. terminalis* leaves (273.89 ± 0.6 μg/g DW) followed by *B. terminalis* stem (4.13 ± 0.1 μg/g DW) (Figure 5). Similarly, remarkable quantity of verbascoside was observed in *C. grandiflorus* stem (2.06 ± 0.1 μg/g DW) and leaves (1.56 ± 0.1 μg/g DW) (Figure 5). Compounds detected in extracts of *B. terminalis* and *C. grandiflorus* leaves by LC-MS are shown in Table 4 and Figures 6A,B. Likewise, using GC-MS several bioactive constituents were identified from the methanol extracts of both the species (Table 5). All the compounds from the both species were identified on the basis of chromatogram area, peak, molecular weight and molecular formula.

**TABLE 4:** Compounds detected from the leaf extracts of *B. terminalis* and *C. grandiflorus* by LC-MS.

Group	Name	Molecular formula	RT	m/z	Mass
Phenolics	Catechin-4beta-ol^a^	C_15_H_14_O_7_	3.82	305.0677	306.075
	Hydroquinone^a^	C_6_H_6_O_2_	10.91	109.0295	110.0369
	Salicylic acid^a^ ^,^ ^b^	C_7_H_6_O_3_	12.77	137.024	138.0313
	Maritimetin^a^	C_15_H_10_O_6_	20.16	285.0394	286.0467
	Sinapyl aldehyde^a^	C_11_H_12_O_4_	21.31	207.0656	208.073
	4-Hydroxystyrene^b^	C_8_H_8_O	15.95	119.0501	120.0574
	Fraxin^a^	C_16_H_18_O_10_	3.59	369.0829	370.0901
Glycoside	Antirrhinoside^a.b^	C_15_H_22_O_10_	4.21	361.1125	362.1198
	Swertiamarin^b^	C_16_H_22_O_10_	8.2	373.1133	374.1208
	Shanzhiside^b^	C_16_H_24_O_11_	6.84	391.1237	392.131
Terpene glycoside	Leonuridine^b^	C_15_H_24_O_9_	8.47	348.1409	348.1409
Steroid	Dexamethasone Acetate^a^	C_24_H_31_FO_6_	13.61	433.2042	434.2115
	Hydroxyanthraquinone^a^	C_14_H_8_O_3_	25.47	223.0397	224.0469
Anthraquinone	2-Hydroxymethylanthraquinonea^a^	C_15_H_10_O_3_	27.98	237.0556	238.0628
Xanthonoid	Gambogic acid^a^	C_38_H_44_O_8_	31.16	627.2974	628.3046
Ethyl ester	Trinexapac-ethyl^a^	C_13_H_16_O_5_	25.36	251.0921	252.0994
Quinoline	Quinolin-2-ol^a^	C_9_H_7_NO	18.46	144.0452	145.0525
Ketone	Zingerone^a^	C_11_H_14_O_3_	25.7	193.0869	194.0941
Tertiary alcohol	Ancymidol^b^	C_15_H_16_N_2_O_2_	18.53	255.1127	256.12
Iridoid monoterpenoid	Monotropein^b^	C_16_H_22_O_11_	9.83	389.1077	390.1149

a Compounds detected in B. terminalis.

b Compounds detected in C. grandiflorus.

**TABLE 5:** Compounds detected from the leaves extracts of *B. terminalis* and *C. grandiflorus* by GC-MS.

Peak no	Group	Name	Molecular formula	RT	^a^Area (%)	^b^Area (%)
1	Phenol	Phenol, 2,4-bis(1,1-dimethylethyl)^a^	C_17_H_30_OSi	16.764	0.84	ND
2	Phthalate ester	Diethyl Phthalate^b^	C_12_H_14_O_4_	17.76	ND	3.21
3	Fatty acid	Tetradecanoic acid^a^ ^,^ ^b^	C_14_H_28_O_2_	19.559	5.54	0.91
4	Diterpene	Neophytadiene^a^	C_20_H_38_	20.328	0.91	ND
7	Fatty acid	n-Hexadecanoic acid^a^ ^,^ ^b^	C_16_H_32_O_2_	21.597	20.45	19.31
8	Diterpene alcohol	Phytol^a^	C_20_H_40_O	23.026	4.40	ND
11	Fatty acid	Octadecanoic acid^a^ ^,^ ^b^	C_18_H_36_O_2_	23.301	5.71	11.45
22	Triterpene	Squalene^b^	C_30_H_50_	28.74	ND	3.84
23	Fat soluble compound	Vitamin E^b^	C_29_H_50_O_2_	32.22	ND	0.77
25	Phytosterol	Stigmasterol^b^	C_29_H_48_O	34.42	ND	2.85

a Compounds detected in B. terminalis.

b Compounds detected in C. grandiflorus, ND- not detected.

## 4 Discussion

This investigation reported reliable amount of phenolics, flavonoids, tannins, proanthocyanidins and iriodoids from the leaves and stem of both the species. Our findings support the results of Amoo et al. (2011) who observed significant amount of phenolics, flavonoids, tannins, proanthocyanidins and iridoid content in methanolic extract leaves, stem and root of three *Barleria* species. Antioxidant potential of leaves and stem of *B. terminalis* and *C. grandiflorus* were comparable to the findings of Amoo et al. (2011) and Yadav et al. (2012). Extraction of phytochemicals depends on their solubility in different solvents and hence, the variability (quantitative and qualitative) in phytochemicals could be on account of different solvents used. Also, the extraction is dependent upon the extraction process, the genotype, and many intrinsic and extrinsic factors (Attar and Ghane, 2019).

Amoo et al. (2011) reported that methanol extract of leaves *B. greenii* and *B. albostellata* showed promising inhibition of AChE (68 and 22%, respectively). Investigations of *α*-amylase and *α*-glucosidase inhibitory activity are the key steps to discover the plants with antidiabetic potential as they play important role in the cure of diabetes (Ghane et al., 2018; Attar and Ghane, 2019; Patel et al., 2020; Taslimi et al., 2020). In the present investigation, correlation among phytochemicals and antioxidant activities from both the leaves and stem extracts of various solvents of both the species was found by PCA. Acetone was found to be the most superior for extracting phytochemicals. Acetone extracts showed higher antioxidant activity than ethanol, methanol and water extracts. This solvent can be used for the extraction of natural antioxidants from both the species.

HPLC analysis confirmed the presence of potent phenolics and phenylethanoid glycoside (verbascoside). Bioactive compounds found in plants also play important role in pharmaceutics and food industry, where some secondary metabolites showed potential biological activities (Nescatelli et al., 2017; Stefanucci et al., 2018; Uysal et al., 2019; Lekhak et al., 2021; Mollica et al., 2021; Patel and Ghane, 2021). Ranade et al. (2016) concluded that among phenolics, GA and CA were found the most common and abundant in leaves and stem of *B. prionitis*. Verbascoside was firstly isolated from the Mullein plant (*Verbascum sinuatum* L.). It has potent biological activities such as antioxidant, anti-inflammatory, antineoplastic, wound healing, neuroprotective, etc. (Alipieva et al., 2014). Kanchanapoom et al. (2004) isolated verbascoside from the methanolic extract of entire plant of *B. strigosa*. Additionally, LC-MS and GC-MS analysis revealed several potent metabolites from the methanolic extract of both species. Studies on phytochemical investigations supported appreciable bioactivities from the studied plants coupled with the presence of bioactive compounds.

## 5 Conclusion

The presence of considerable amount of phenolics, flavonoids, tannins, terpenoids, proanthocyanidins and iridoids in *B. terminalis* and *C. grandiflorus* led to appreciable antioxidant, antidiabetic, and anti-acetyl cholinesterase potential. Acetone was discovered to be the best solvent for extracting phytochemicals that exhibited potential antioxidant properties in the current study. Ethanol extracts of leaves and stem revealed promising antidiabetic and acetyl cholinesterase inhibitory activities. The presence of phenolics, verbascoside, and other bioactive substances were confirmed using RP-HPLC, LC-MS, and GC-MS. The best source of phenolics and phenylethanoid glycoside was discovered to be *B. terminalis* leaves. Biological activities and secondary metabolites differ from one organ to the other as well as from one season to the next. As India is home to 30 species of *Barleria,* it would be worthwhile to undertake extensive research to look into the phytochemical diversity in the genus and identify potential bioactive compounds that might be used to treat diabetes, neurological disorders, and produce new medications. In addition, the pharmaceutical industry needs to pay more attention to the creation of important pharmaceuticals (phenolics, iridoids, and phenylethanoid glycosides) from *Barleria*.

## Data Availability

The raw data supporting the conclusion of this article will be made available by the authors, without undue reservation.

## Abbreviations

ABTS, (2, 2′-azinobis 3-ethylbenzthiazoline-6-sulfonic acid); AChE, acetylcholinesterase; CHLA, chlorogenic acid; COA, coumaric acid; DPPH, 1,1-diphenyl-2-picrylhydrazyl; DNSA, dinitrosalicylic acid; DTNB, 5, 5-dithiobis-2-nitrobenzoic acid; DW, distilled water; FRAP, ferric reducing antioxidant property; GA, gallic acid; GC-MS, gas chromatography-mass spectrometry; HBA, hydroxybenzoic acid; HR-LC-MS, high-resolution liquid chromatography-mass spectrometry; MCA, metal chelating activity; PMA, phosphomolybdenum activity; PCA, principal component analysis; RP-HPLC, reversed phase high performance liquid chromatography; TIC, total iridoid content; TFC, total flavonoids content; TPC, total phenolic content; TPTZ-HCl, 2,4,6-Tripyridyl-S-triazine- hydrochloric acid; TTC, total tannin content; VA, vanillic acid.

## References

[B1] Alipieva K. Korkina L. Orhan I. E. Georgiev M. I. (2014). Verbascoside-a Review of its Occurrence, (Bio)synthesis and Pharmacological Significance. Biotechnol. Adv. 32, 1065–1076. 10.1016/j.biotechadv.2014.07.001 25048704

[B2] Amoo S. O. Ndhlala A. R. Finnie J. F. Van Staden J. (2011). Antifungal, Acetylcholinesterase Inhibition, Antioxidant and Phytochemical Properties of Three *Barleria* Species. South Afr. J. Bot. 77, 435–445. 10.1016/j.sajb.2010.11.002

[B3] Ata A. Kalhari K. S. Samarasekera R. (2009). Chemical Constituents of *Barleria Prionitis* and Their Enzyme Inhibitory and Free Radical Scavenging Activities. Phytochemistry Lett. 2, 37–40. 10.1016/j.phytol.2008.11.005

[B4] Attar U. A. Ghane S. G. (2019). *In Vitro* antioxidant, Antidiabetic, Antiacetylcholine Esterase, Anticancer Activities and RP-HPLC Analysis of Phenolics from the Wild Bottle Gourd (*Lagenaria siceraria* (Molina) Standl.). South Afr. J. Bot. 125, 360–370. 10.1016/j.sajb.2019.08.004

[B5] Attar U. A. Ghane S. G. (2021). Proximate Composition, Ionomics, Phytochemical, Antioxidant, Anti-diabetic and Acetylcholinesterase Inhibitory Activity of *Cucumis* Species from Western Ghats of India. Indian J. Pharm. Sci. 83 (4), 679–694. 10.36468/pharmaceutical-sciences.819

[B6] Banerjee D. Maji A. K. Mahapatra S. Banerji P. (2012). *Barleria prionitis* Linn.: A Review of its Traditional Uses, Phytochemistry, Pharmacology and Toxicity. Res. J. Phytochem. 6, 31–41. 10.3923/rjphyto.2012.31.41

[B7] Benzie I. F. Strain J. J. (1996). The Ferric Reducing Ability of Plasma (FRAP) as a Measure of “Antioxidant Power”: the FRAP Assay. Anal. Biochem. 239, 70–76. 10.1006/abio.1996.0292 8660627

[B8] Darbyshire I. Tripp E. A. Chase F. M. (2019). A Taxonomic Revision of Acanthaceae Tribe Barlerieae in Angola and Namibia. Part 1. Kew Bull. 74 (5), 1–85. 10.1007/s12225-018-9791-0

[B9] Dhakulkar S. Ganapathi T. R. Bhargava S. Bapat V. A. (2005). Induction of Hairy Roots in *Gmelina arborea* Roxb. And Production of Verbascoside in Hairy Roots. Plant Sci. 169, 812–818. 10.1016/j.plantsci.2005.05.014

[B10] Fawole O. A. Ndhlala A. R. Amoo S. O. Finnie J. F. Van Staden J. (2009). Anti-inflammatory and Phytochemical Properties of Twelve Medicinal Plants Used for Treating Gastro-Intestinal Ailments in South Africa. J. Ethnopharmacol. 123, 237–243. 10.1016/j.jep.2009.03.012 19429367

[B11] Ghane S. G. Attar U. A. Yadav P. B. Lekhak M. M. (2018). Antioxidant, Anti-diabetic, Acetylcholinesterase Inhibitory Potential and Estimation of Alkaloids (Lycorine and Galanthamine) from *Crinum* Species: An Important Source of Anticancer and Anti-alzheimer Drug. Ind. Crops Prod. 125, 168–177. 10.1016/j.indcrop.2018.08.087

[B12] Kanchanapoom T. Noiarsa P. Ruchirawat S. Kasai R. Otsuka H. (2004). Phenylethanoid and Iridoid Glycosides from the Thai Medicinal Plant, *Barleria strigosa* . Chem. Pharm. Bull. 52 (5), 612–614. 10.1248/cpb.52.612 15133217

[B13] Kavitha K. Sujatha K. Manoharan S. (2016). Antidiabetic Potential of Acanthaceae Family. Int. J. Pharm. Sci. Rev. Res. 36 (1), 30–37.

[B14] Lekhak M. M. Patel S. B. Otari S. S. Lekhak U. M. Ghane S. G. (2021). Bioactive Potential and RP-HPLC Detection of Phenolics and Alkaloids (Lycorine and Galanthamine) from Ultrasonic-Assisted Extracts of *Crinum* Roots. South Afr. J. Bot. 10.1016/j.sajb.2021.07.024

[B15] Levieille G. Wilson G. (2002). *In Vitro* propagation and Iridoid Analysis of the Medicinal Species *Harpagophytum procumbens* and *H . zeyheri* . Plant Cell Rep. 21, 220–225. 10.1007/s00299-002-0520-6

[B16] Mollica A. Scioli G. Della Valle A. Cichelli A. Novellino E. Bauer M. (2021). Phenolic Analysis and *In Vitro* Biological Activity of Red Wine, Pomace and Grape Seeds Oil Derived from *Vitis vinifera* L. Cv. Montepulciano d’Abruzzo. Antioxidants 10, 2–17. 10.3390/antiox10111704 PMC8615145 34829574

[B17] Nescatelli R. Carradori S. Marini F. Caponigro V. Bucci R. De Monte C. (2017). Geographical Characterization by MAE-HPLC and NIR Methodologies and Carbonic Anhydrase Inhibition of Saffron Components. Food Chem. 221, 855–863. 10.1016/j.foodchem.2016.11.086 27979284

[B18] Patel S. B. Ghane S. G. (2021). Phyto-constituents Profiling of *Luffa echinata* and *In Vitro* Assessment of Antioxidant, Anti-diabetic, Anticancer and Anti-acetylcholine Esterase Activities. Saudi J. Biol. Sci. 28 (7), 3835–3846. 10.1016/j.sjbs.2021.03.050 34220238 PMC8241619

[B19] Patel S. B. Attar U. A. Ghane S. G. (2018). Antioxidant Potential of Wild *Lagenaria siceraria* (Molina) Standl. Thai J. Pharm. Sci. 42, 90–96.

[B20] Patel S. B. Attar U. A. Sakate D. M. Ghane S. G. (2020). Efficient Extraction of Cucurbitacins from *Diplocyclos Palmatus* (L.) C. Jeffrey: Optimization Using Response Surface Methodology, Extraction Methods and Study of Some Important Bioactivities. Sci. Rep. 10, 2109–2112. 10.1038/s41598-020-58924-5 32034276 PMC7005863

[B21] Patil S. S. Tamboli A. S. Yadav S. R. Lekhak M. M. (2019). A New Species of *Barleria* (Acanthaceae), its Morphotaxonomy, Cytogenetics and Phylogenetic Placement. Plant Syst. Evol. 305, 933–947. 10.1007/s00606-019-01613-2

[B22] Patil S. S. Ganesan R. Yadav S. R. Lekhak M. M. (2020). Taxonomy of *Barleria morrisiana* E. Barnes & C. E. C. Fisch. (Acanthaceae), a Little-Known Species of the Biligiri Ranganathaswamy temple Tiger reserve, Karnataka, India. Curr. Sci. 119 (10), 1611–1612.

[B23] Prieto P. Pineda M. Aguilar M. (1999). Spectrophotometric Quantitation of Antioxidant Capacity through the Formation of a Phosphomolybdenum Complex: Specific Application to the Determination of Vitamin E. Anal. Biochem. 269, 337–341. 10.1006/abio.1999.4019 10222007

[B24] Ranade R. Jain A. Joshi N. (2016). Estimation of Phenolic Compounds by RP-HPLC and Antioxidant Activity in Leaf and Stem Extracts of *Barleria prionitis* L. Int. J. Pharm. Sci. Res. 7 (6), 2445–2457. 10.13040/IJPSR.0975-8232.7(6).2445-57

[B25] Sakanaka S. Tachibana Y. Okada Y. (2005). Preparation and Antioxidant Properties of Extracts of Japanese Persimmon Leaf tea (Kakinoha-Cha). Food Chem. 89, 569–575. 10.1016/j.foodchem.2004.03.013

[B26] Stefanucci A. Mollica A. Macedonio G. Zengin G. Ahmed A. A. Novellino E. (2018). Exogenous Opioid Peptides Derived from Food Proteins and Their Possible Uses as Dietary Supplements: A Critical Review. Food Rev. Int. 34 (1), 70–86. 10.1080/87559129.2016.1225220

[B27] Sudheer W. N. Praveen N. (2021). Phytochemical, Pharmacological and Tissue Culture Studies of Some Important Species of the Genus *Barleria* L. (Acanthaceae) - a Review. Plant Sci. Today 8 (3), 491–500. 10.14719/pst.2021.8.3.1117

[B28] Taslimi P. Köksal E. Gören A. C. Bursal E. Aras A. Kılıç Ö. (2020). Anti-alzheimer, Antidiabetic and Antioxidant Potential of *Satureja cuneifolia* and Analysis of its Phenolic Contents by LC-MS/MS. Arabian J. Chem. 13, 4528–4537. 10.1016/j.arabjc.2019.10.002

[B29] Tripp E. A. Daniel T. F. Fatimah S. McDade L. A. (2013). Phylogenetic Relationships within Ruellieae (Acanthaceae) and a Revised Classification. Int. J. Plant Sci. 174 (1), 97–137. 10.1086/668248

[B30] Uysal A. Ozer O. Y. Zengin G. Stefanucci A. Mollica A. Picot-Allain C. M. N. (2019). Multifunctional Approaches to Provide Potential Pharmacophores for the Pharmacy Shelf: *Heracleum sphondylium* L. subsp. *ternatum* (Velen.) Brummitt. Comput. Biol. Chem. 78, 64–73. 10.1016/j.compbiolchem.2018.11.018 30500554

[B31] Yadav S. A. Raj A. J. Sathishkumar R. (2012). *In Vitro* antioxidant Activity of *Barleria noctiflora* L. F. Asian Pac. J. Trop. Biomed. 2, 716–722. 10.1016/S2221-1691(12)60302-5

